# Stearic Acid/Inorganic Porous Matrix Phase Change Composite for Hot Water Systems

**DOI:** 10.3390/molecules24081482

**Published:** 2019-04-15

**Authors:** Ling Xu, Rui Yang

**Affiliations:** Department of Chemical Engineering, Tsinghua University, Beijing 100084, China; xu-l16@mails.tsinghua.edu.cn

**Keywords:** stearic acid, inorganic porous matrix, expanded graphite (EG), absorption ability, dynamic response, long-term cyclic stability

## Abstract

The storage and utilization of waste heat in low and medium temperature ranges using phase change materials (PCMs) is an effective technology to improve energy utilization efficiency in combined cooling, heating, and power (CCHP) systems. In this paper, stearic acid/inorganic porous matrix phase change composites were developed to store waste heat for hot water systems. Among them, stearic acid/expanded graphite (EG) phase change composite was highlighted and the thermal physical properties, the dynamic response, and the long-term cyclic stability were evaluated. The stearic acid concentrations in the composites were over 95 wt%. The thermal diffusion coefficients were 3–5 times higher than pure stearic acid, independent of composite densities. Accordingly, the heat storage and release times were decreased by up to 41% and 55%, respectively. After 100 cycles, the composites maintained good dynamic response and long-term cyclic stability, with heat storage density of 122–152 MJ/m^3^. Hence, this stearic acid/EG phase change composite exhibits excellent comprehensive performances. It is also easy to be prepared and flexible for various types of heat exchangers.

## 1. Introduction

Combined cooling, heating, and power systems (CCHP) are efficient and eco-friendly energy systems with reduced energy consumption and greenhouse gas emissions. By introducing energy storage units (ESUs) into CCHP systems, the energy mismatch between the supply from CCHP systems and the demand of users could be alleviated and the CCHP systems are able to operate stably with reduced capacities [1,2,3]. Phase change materials, with high thermal storage density and isothermal operation, are expected to be used as short-term storage in CCHP systems to further improve the energy efficiency by the rational distribution of thermal energy in time and space.

Stearic acid, with the phase change temperature about 70 °C, has high latent heat, excellent thermal stability, and little super-cooling. It is suitable to store thermal energy (like waste heat, solar energy, etc.) as an accessory energy storage unit in hot water systems, which can bridge the gap between the energy resources, hot water systems, and users to meet energy demands at all times [4,5,6,7,8]. However, the low thermal conductivity of stearic acid and its volume change during the solid-liquid phase change process will cause a dull dynamic response to an environment outside and with unstable heat transfer, respectively. Therefore, it is essential to take some measures to enhance the thermal conductivity and shape stability of stearic acid for industrial application.

Two conventional methods are used to overcome the low thermal conductivity and volume change of stearic acid: (1) Microencapsulating stearic acid as core material by different kinds of shells such as carbon materials [9,10,11], SiO_2_ [12], and montmorillonite (MMT) [13]. Dao and Jeong [10] reported on the effect of ultra-thin graphene shell on the shape stability and thermal conductivity of stearic acid. The results showed that stearic acid was entrapped well with around 1 wt% graphene and the thermal conductivity of the composite was enhanced due to the thermally conductive graphene. Lin et al. [12] introduced graphene oxide on the surface of microencapsulated stearic acid/silica and found that the encapsulation efficiency reached 83.33% and the thermal conductivity was increased by 75%, compared with pure stearic acid. Yi et al. [13] proposed a core-shell MMT/stearic acid composite with more than 80% stearic acid inside and the thermal conductivity was achieved with a 159.46% increase by MMT nanosheets. (2) Absorbing stearic acid into porous materials such as carbon materials, metal foams, dioxides, clays, expanded vermiculite (EV), and so on [14,15,16,17,18,19,20,21,22,23,24]. For example, Li et al. [16] investigated the effects of a carbonized 3D reduced graphene/metal-organic framework on the thermal energy storage performance of stearic acid. The results revealed that stearic acid was well stabilized into the porous skeleton via capillary forces and physical interactions between them and the thermal conductivity of the composite was improved up to 27.7% with an increase in phase change latent heat. Wu et al. [17] prepared stearic acid/EG composites and compressed them into stable-shape block. Stearic acid was dispersed well into graphite flakes with no super-cooling and the thermal conductivities at axial and racial directions were enhanced significantly. The maximum loss of stearic acid was less than 0.5% for stearic acid/25% EG with a packed density of 900 kg/m^3^ during long-term thermal cycles. Zhang et al. vacuum impregnated stearic acid into porous carbonized expanded vermiculite [18]. The results suggested that 63.12% of PCM was retained in the EVC without leakage and the thermal conductivity was obtained an increase of 52.9% compared with that of the stearic acid/EV composite.

Herein, stearic acid was directly absorbed by EGs expanded at different temperatures, organic montmorillonite (OMMT), attapulgite, expanded perlite and sepiolite, respectively. Because of excellent absorption ability, stearic acid/EG composites were finally chosen for further research on thermal energy storage performance. The heat storage and release characterization of the composites were highly strengthened and the thermal physical properties were maintained well during long-term thermal cycles. Therefore, this stearic acid/EG phase change composite presented excellent comprehensive performances and had the potential to be applied to various types of heat exchanger.

## 2. Results and Discussion

### 2.1. Absorption Ability

**Figure 1:**
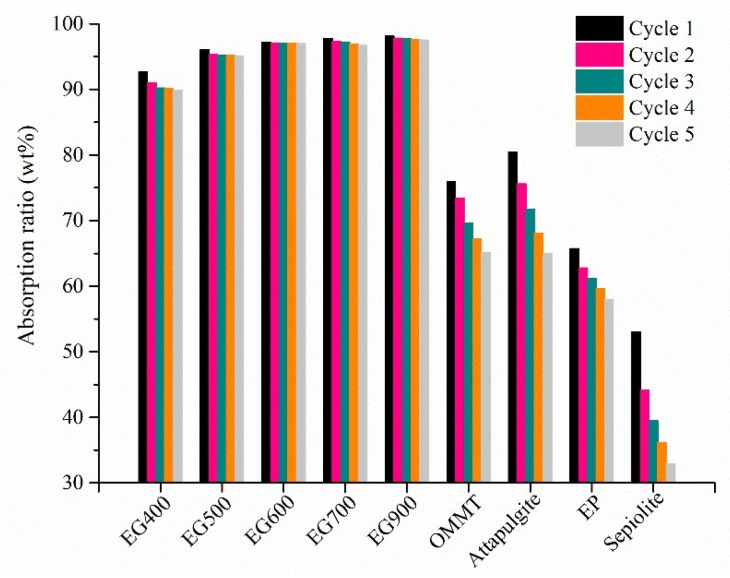
Absorption ratios of different inorganic porous skeletons to stearic acid during five cycles.

Figure 1 shows absorption ratios of different inorganic skeletons with stearic acid during five cycles. It was seen that EGs had absorption ratios of over 90 wt%, which were much larger than OMMT, attapulgite, expanded perlite, and sepiolite. As the expansion temperature increased, the absorption ratio of EG increased slightly. After five cycles, the absorption ratios of EG500, EG600, EG700, and EG900 maintained above 95 wt%, whereas the absorption ratios of OMMT, attapulgite, EP, and sepiolite decreased, showing poor absorption ability.

### 2.2. Microstructure

**Figure 2:**
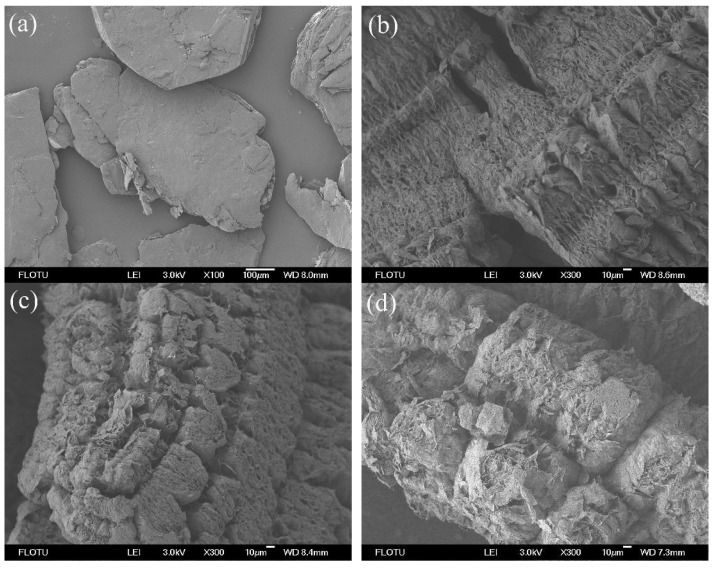

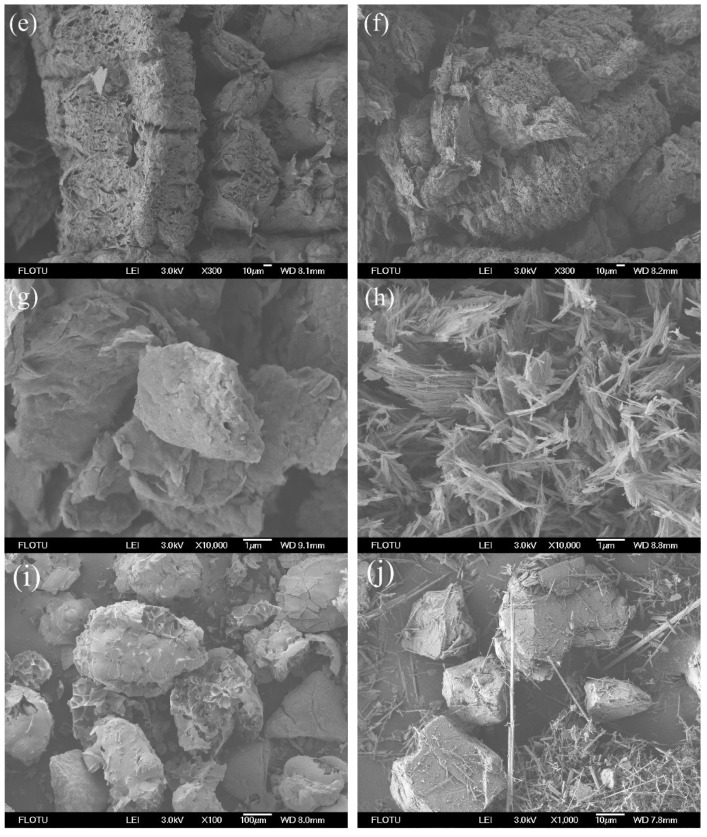
Field Emission Scanning Electron Microscopy (FESEM) micrographs of inorganic porous skeletons: (**a**) Expandable graphite, (**b**) EG400, (**c**) EG500, (**d**) EG600, (**e**) EG700, (**f**) EG900, (**g**) organic montmorillonite (OMMT), (**h**) attapulgite, (**i**) EP, and (**j**) sepiolite.

Figure 2 shows the microstructures of different inorganic skeletons. Expandable graphite appeared as a long flat structure with no absorption ability. After expansion at high temperatures, it turned into a porous worm-like structure, which favored good absorption of stearic acid. OMMT was a massive structure with lamellar structure in bulk, whereas attapulgite was an acicular aggregated structure, and sepiolite was a fibrous or massive structure composed of stacked filaments. The three inorganic skeletons had poor absorption ability with gradually decreased absorption ratios during cycles, compared to the porous EG. Expanded perlite was a cellular structure and also had poor absorption ability due to its large pore size.

### 2.3. Pore Size Distribution

**Figure 3:**
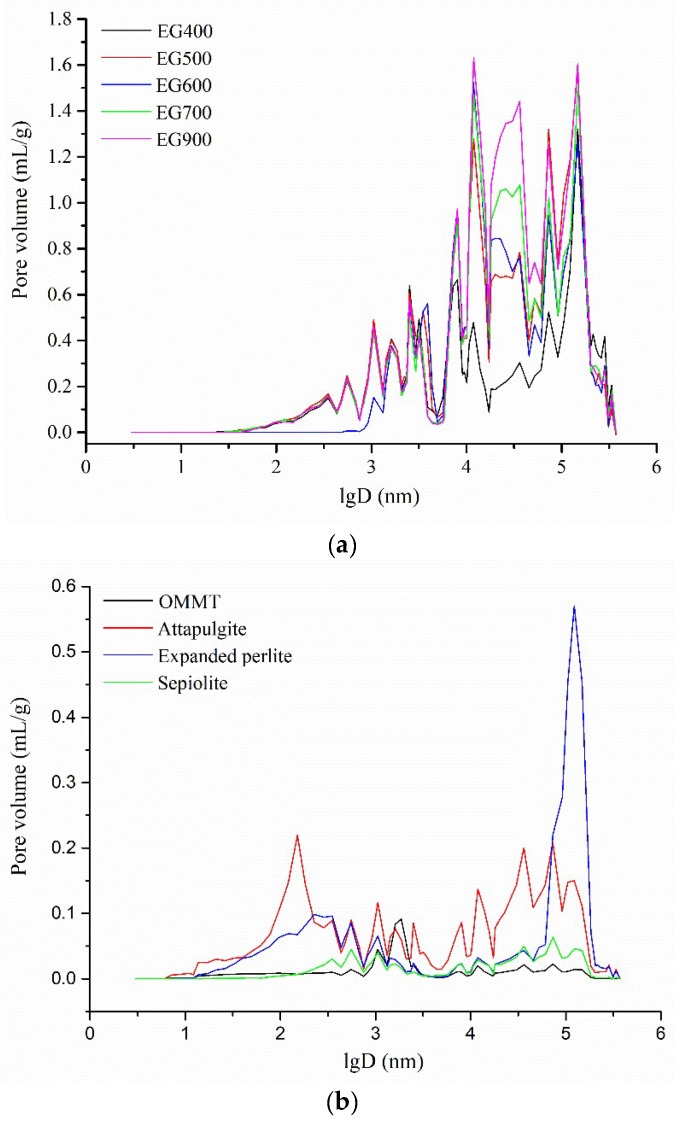
Pore size distribution of (**a**) EG treated at different temperatures, (**b**) other inorganic skeletons.

Figure 3 shows the pore size distributions of EGs treated at different temperatures and other inorganic skeletons. Obviously, the pore volumes of EGs were much larger than those of OMMT, attapulgite, EP, and sepiolite within the pore diameter range, and so exhibited better absorption ability than them. In addition, the higher the expansion temperature, the larger the pore volumes of EG, thus the absorption capacity of EG was increased with the increasing expansion temperature, despite the same pore size distribution.

### 2.4. Thermal Diffusion Coefficient

**Figure 4:**
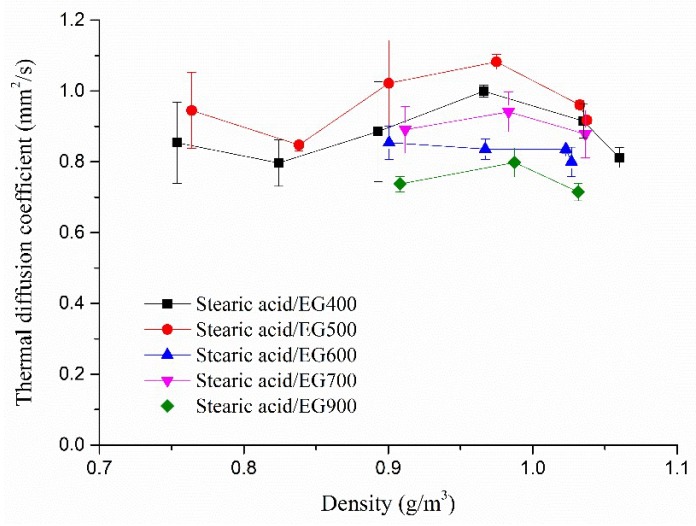
The thermal diffusion coefficients of stearic acid/EG composites with different densities.

Due to the excellent absorption of EG to stearic acid, a stearic acid/EG phase change composite was chosen for further research on dynamic response and long-term thermal stability. Figure 4 shows the thermal diffusion coefficients of stearic acid/EG phase change composites with different densities. The thermal diffusion coefficient of stearic acid was about 0.2 mm^2^/s. After being absorbed by EG, the thermal diffusion coefficients of stearic acid/EG composites were increased to between 0.65 and 1.10 mm^2^/s, which were 3–5 times higher than that of pure stearic acid. It should be noted that the high density of stearic acid/EG composite would destroy the pore structure of EG and thus weaken its absorption ability to stearic acid. However, Figure 4 suggests that the thermal diffusion coefficient of stearic acid/EG composite was little dependent on the bulk density. That is, the thermal conductivity of stearic acid/EG composite could be improved without destruction of the pore structure of EG with relative low density.

### 2.5. Dynamic Response and Long-Term Cycle Stability

**Figure 5:**
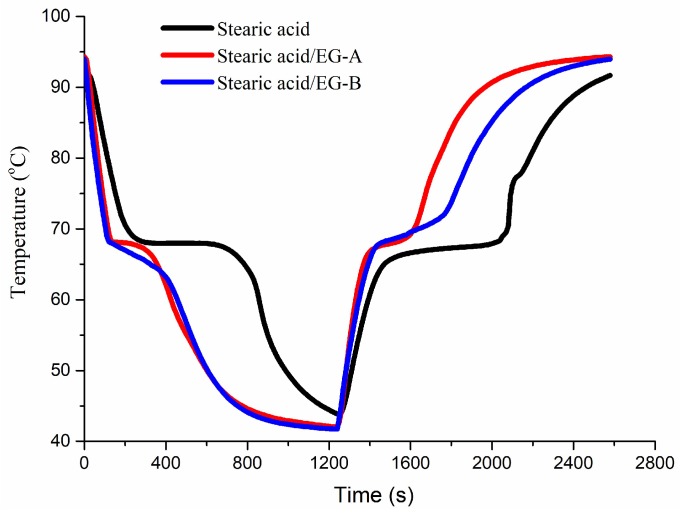
The thermal storage and release curves of stearic acid and stearic acid/EG composites.

**Figure 6:**
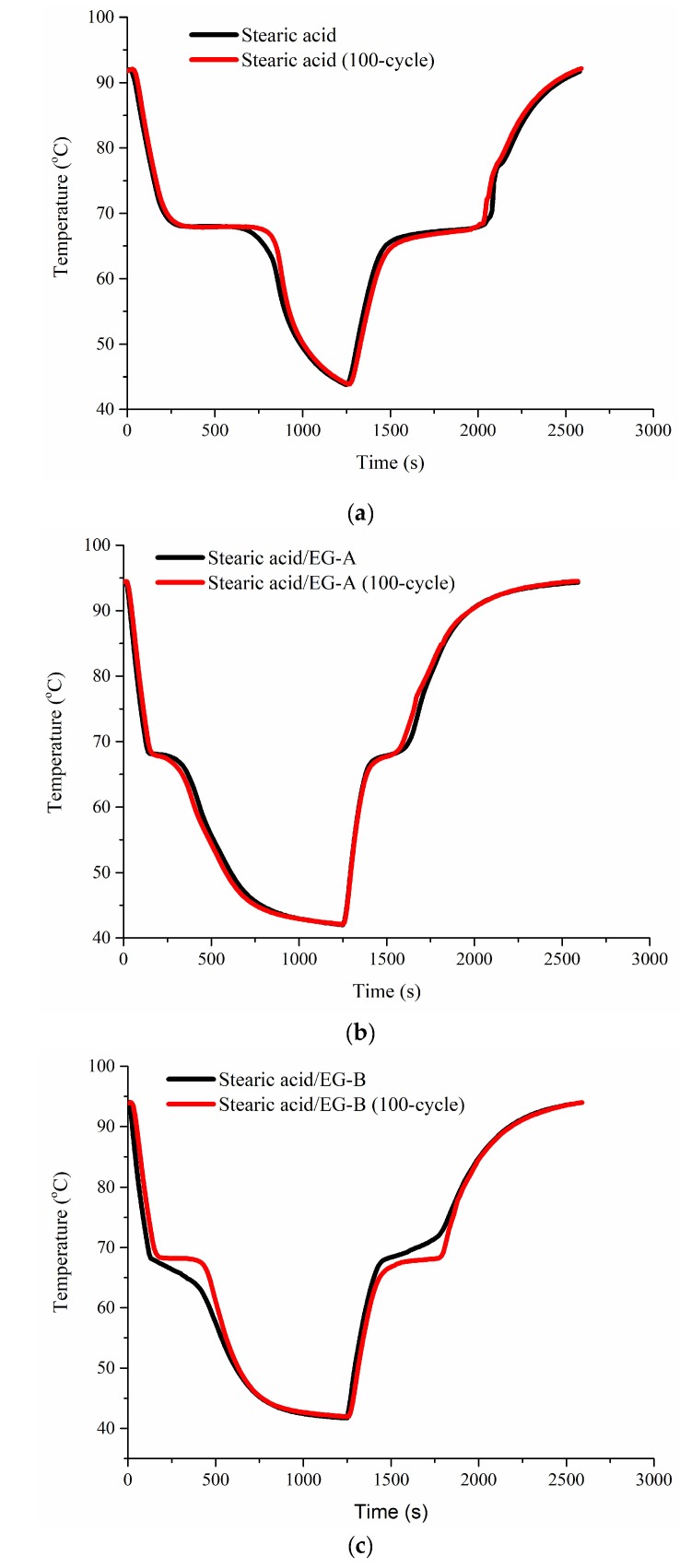
The thermal storage and release curves before and after 100 cycles: (**a**) Stearic acid, (**b**) stearic acid/EG-A, and (**c**) stearic acid/EG-B.

To minimize the leakage of stearic acid during thermal cycles, a stearic acid/EG composite with a mass ratio of 95/5 was developed to investigate the dynamic response and long-term cycle stability. Stearic acid/EG was filled into two stainless steel cubes with two packed densities. The density of stearic acid/EG-A was 0.63 g/cm³ and the density of stearic acid/EG-B was 0.75 g/cm³. Their thermal storage and release processes are shown in Figure 5. The thermal storage and release times of stearic acid were both about 580 s. After being incorporated with EG, the thermal storage and release times of stearic acid/EG-A were 210 and 200 s, a decrease of 64% and 66%, respectively. The thermal storage and release times of stearic acid/EG-B were 340 and 260 s, a decrease of 41% and 55%, respectively. Therefore, the dynamic response was enhanced greatly, which was in accordance with the results in Figure 4. Figure 6 shows the thermal storage and release curves of stearic acid and stearic acid/EG after 100 thermal cycles. It was seen that the phase change temperatures of stearic acid and stearic acid/EG composites maintained at 63–69 °C and the thermal storage and release processes after 100 cycles were almost identical to the original ones, which thus confirmed the stable absorption capacity of EG to stearic acid during long-term thermal cycles.

Table 1 and Table 2 show stearic acid and stearic acid/EG before and after 100 cycles were tested using DSC and their thermal physical properties. Stearic acid was stable with little change in phase change temperatures and enthalpies before and after 100 cycles during heating and cooling processes. When absorbed by EG, the melting and freezing temperatures were slightly increased and decreased, respectively. In addition, the phase change enthalpies of stearic acid/EG were reduced to 92–96% of stearic acid, which were almost the same with the calculated values in theory. After 100 cycles, the phase change temperatures of stearic acid/EG showed little changes, and the enthalpy of stearic acid/EG-A was decreased by only about 7%, while the enthalpy of stearic/EG-B remained almost unchanged. Moreover, the thermal storage density of stearic acid/EG was 122–152 MJ/m^3^.

## 3. Experimental

### 3.1. Materials

Stearic acid (97%), with a density of 840 kg/m^3^, was purchased from Acros Organics. Expandable graphite (50 mesh) was provided by Qingdao Tengshengda Tansujixie Co., Ltd. (Qingdao, China). Organic montmorillonite (OMMT, NB901) were purchased from Zhejiang Huate Chemical Co., Ltd. (Zhejiang, China). Attapulgite, expanded perlite, and sepiolite were obtained from Jiangsu Jiuchuan Nano Technology Co., Ltd. (Huaian, China).

### 3.2. Sample Preparation

Expandable graphite was expanded in a muffle furnace at 400 °C, 500 °C, 600 °C, 700 °C, and 900 °C for 2 min, respectively, and the obtained EGs were labeled as EG400, EG500, EG600, EG700, and EG900, respectively.

Porous material (i.e., EGs, OMMT, attapulgite, expanded perlite, and sepiolite) was put into a beaker containing excessive stearic acid and the mixture was placed in an air-circulating oven at 100 °C for 2 h. Next, the mixture was poured into a sieve to remove the remaining stearic acid and then put back in the oven at 100 °C for 2 h. After that, the bottom of the sieve was wiped with absorbent cotton to remove the exuded stearic acid. Finally, the mixture was weighed at room temperature. All the steps were repeated for five cycles and the absorption ratios (the mass of stearic acid to the mass of stearic acid/porous material) were obtained.

### 3.3. Characterization

#### 3.3.1. Field Emission Scanning Electron Microscopy (FESEM)

FESEM was performed on a JEOL model JSM-7401 (JEOL Ltd., Tokyo, Japan) apparatus with an operating voltage of 3.0 kV to investigate the morphology of EGs, OMMT, Attapulgite, expanded perlite, and sepiolite.

#### 3.3.2. Porosity Test

The pore size distributions of EGs, OMMT, Attapulgite, expanded perlite, and sepiolite were characterized by a Mercury analyzer (AutoporeIV9510, Micromeritics instrument (Shanghai) Ltd., Shanghai, China).

#### 3.3.3. Thermal Diffusion Coefficient

Stearic acid/EG (95/5) composites were cold compressed into small cylinders with different densities (φ12.7 mm × 5 mm). The thermal diffusion coefficients (mm^2^/s) of the samples were then measured using the laser flash method (LFA467, NETZSCH Scientific Instruments Trading (Shanghai) Ltd., Shanghai, China) at 20 °C.

#### 3.3.4. Thermal Shock Tester

A stearic acid/EG (95/5) composite was directly filled into a stainless steel cube and then placed in the thermal shock tester (GP/3TS48-20, GP Co., Ltd., Shanghai, China) to undergo heating-cooling cycles. The sample was switched between two chambers set at 95 °C and 40 °C, respectively. The heat release and storage curves were recorded during the cooling and heating processes.

#### 3.3.5. Differential Scanning Calorimetry (DSC)

The phase change temperature and latent heat of a sample were tested using DSC (Q100, TA Instrument, Shanghai, China) in a temperature range of −30–50 °C at 10 °C/min.

## 4. Conclusions

A stearic acid/EG phase change composite was prepared and would be applied in storing waste heat for hot water system. EG had a large pore volume and was able to absorb more than 95 wt% stearic acid. The thermal diffusion coefficient of stearic acid/EG composite was increased from 0.2 to 0.65–1.10 mm^2^/s, independent of composite density. In addition, the dynamic response of stearic acid/EG was greatly enhanced. The thermal storage times of stearic acid/EG with a density of 0.63 and 0.75 g/cm^3^ were reduced from 580 s to 210 s and 340 s, respectively, and the thermal release times were decreased from 580 s to 200 s and 260 s, respectively. Moreover, the absorption ability and thermal physical properties of stearic/EG composites were stable, with constant phase change temperature range of 63–69 °C, little enthalpy changes and almost identical thermal storage and release processes during 100 thermal cycles. Stearic acid/EG composite was easy to be prepared and convenient to fill into various types of heat exchangers used in hot water systems.

## Figures and Tables

**Table 1:** Differential scanning calorimetry (DSC) data of stearic acid and stearic acid/EG composites during heating.

Samples	Heating			
	Before 100 Cycles		After 100 Cycles	
	Melting Temperature (°C)	Enthalpy (J/g)	Melting Temperature (°C)	Enthalpy (J/g)
Stearic acid	72.3	216.5	72.7	218.5
Stearic acid/EG-A	76.1	208.0	77.3	193.1
Stearic acid/EG-B	78.8	202.6	78.5	199.7

**Table 2:** DSC data of stearic acid and stearic acid/EG composites during cooling.

Samples	Cooling			
	0 cycle		After 100 Cycles	
	Freezing Temperature (°C)	Enthalpy (J/g)	Freezing Temperature (°C)	Enthalpy (J/g)
Stearic acid	63.0	223.4	63.1	224.4
Stearic acid/EG-A	60.6	213.3	59.8	198.0
Stearic acid/EG-B	59.5	206.3	60.1	202.8
